# Physicochemical Descriptors in Biodistribution and Clearance of Contrast Agents

**DOI:** 10.1002/adpr.202300036

**Published:** 2023-06-02

**Authors:** Sung Ahn, Benjamin Sanchez-Langeling, Jeong Heon Lee, Maged Henary, Kai Bao, Hak Soo Choi

**Affiliations:** Gordon Center for Medical Imaging, Department of Radiology, Massachusetts General Hospital and Harvard Medical School, 149 13th Street, Boston, MA 02129, USA; Department of Chemistry and Chemical Biology, Harvard University, Cambridge, MA 01238, USA; Gordon Center for Medical Imaging, Department of Radiology, Massachusetts General Hospital and Harvard Medical School, 149 13th Street, Boston, MA 02129, USA; Department of Chemistry and Center of Diagnostics and Therapeutics, Georgia State University, 100 Piedmont Avenue SE, Atlanta, GA 30303, USA; Gordon Center for Medical Imaging, Department of Radiology, Massachusetts General Hospital and Harvard Medical School, 149 13th Street, Boston, MA 02129, USA; Gordon Center for Medical Imaging, Department of Radiology, Massachusetts General Hospital and Harvard Medical School, 149 13th Street, Boston, MA 02129, USA

**Keywords:** biodistribution, contrast agents, machine learning, molecular descriptors, physicochemical properties

## Abstract

Hepatobiliary and renal excretions are the two major in vivo elimination pathways, of which prediction is particularly important for the optimization of systemic and/or target-site exposure of new near-infrared (NIR) fluorescent contrast agents. To characterize the physicochemical descriptors associated with each clearance route, a compound library containing more than 760 NIR fluorophores is analyzed and systematically reviewed in this study. Although there are a host of biochemical and physiological mechanisms that ultimately determine the in vivo fate of any given molecule, the physicochemical properties of contrast agents including molecular weight, hydrophobicity, topological polar surface area, molecular charges, and hydrogen/rotatable bonds are often indicative of each clearance route. The approach developed is anticipated to be useful in early lead identification studies when selecting NIR fluorescent contrast agents from large database screenings. It may also be applied to prioritize synthetically feasible chemical modifications during lead compound optimization.

## 1. Introduction

Near-infrared (NIR) imaging is a quickly developing section of medical imaging that offers high resolution, deep tissue penetration, and specific tissue/disease targeting.^[1–3]^ Due to the low autofluorescence of biological tissues in the NIR window, NIR fluorescent contrast agents can provide an ideal signal-to-background ratio (SBR) during image-guided interventions.^[4]^ Currently, two of the most pressing challenges facing bioimaging are the nonspecific uptake of intravenously administered contrast agents and the incomplete elimination of unbound agents from the body. On top of the 4S criteria (high sensitivity, specificity, selectivity, and safety), designing a targeted NIR fluorescent contrast agent that shows improved targetability, suitable clearance, and maximum elimination from the background tissues and eventually the body after completion of targeting is an important consideration and key to an optimized SBR.^[5]^

The typical method for creating targeted NIR contrast agents requires 1) covalent conjugation of separate targeting ligand(s) and an imaging domain or 2) a single compact molecule that performs both targeting and imaging using the structure-inherent targeting (SIT) strategy.^[6]^ Unfortunately, as a part of this systematic discovery, the process of developing targeted contrast agents becomes combinatorically lengthy due to the diversity of the chemical structure, the difficulty of chemical synthesis and purification, and the in vivo evaluation of these fluorophores.^[7]^ Specifically, the absorption, distribution, metabolism, and elimination (ADME) profile of a contrast agent is predominantly governed by the physicochemical properties of the integrated molecule including molecular weight (MW), overall hydrophobicity (logD at pH 7.4), topological polar surface area (TPSA), and surface charges and are not entirely nullified by traditional conjugation to a targeting ligand or using the SIT strategy.^[5,8]^ Similar to drug development, the prediction or measurement of the physicochemical properties at an early phase of contrast agent discovery is crucial to reduce attrition rates due to poor biopharmaceutical properties.^[9–15]^

At present, the in silico quantitative structure–activity (affinity) relationship (QSAR) model has been widely applied to predict the in vivo fate of drugs in the early stage of drug discovery.^[16,17]^ This method allows for improved drug design by creating a target physicochemical “region” but heavily relies on the size of a dataset. To avoid this caveat, for the first time, we compiled a large, diverse, and standardized compound library of more than 760 analogs of common fluorophore motifs including but not limited to polymethines, phenoxazines, and squaraines, in the process of designing targeted contrast agents. Using this database, we summarize the physicochemical descriptors of polymethine NIR fluorophores, especially pentamethines and heptamethines for structure-inherent biodistribution and clearance.

## 2. Results and Discussion

### 2.1. Inevitable Patterns in the Database Reveal Large Diversity

**Figure 1.**
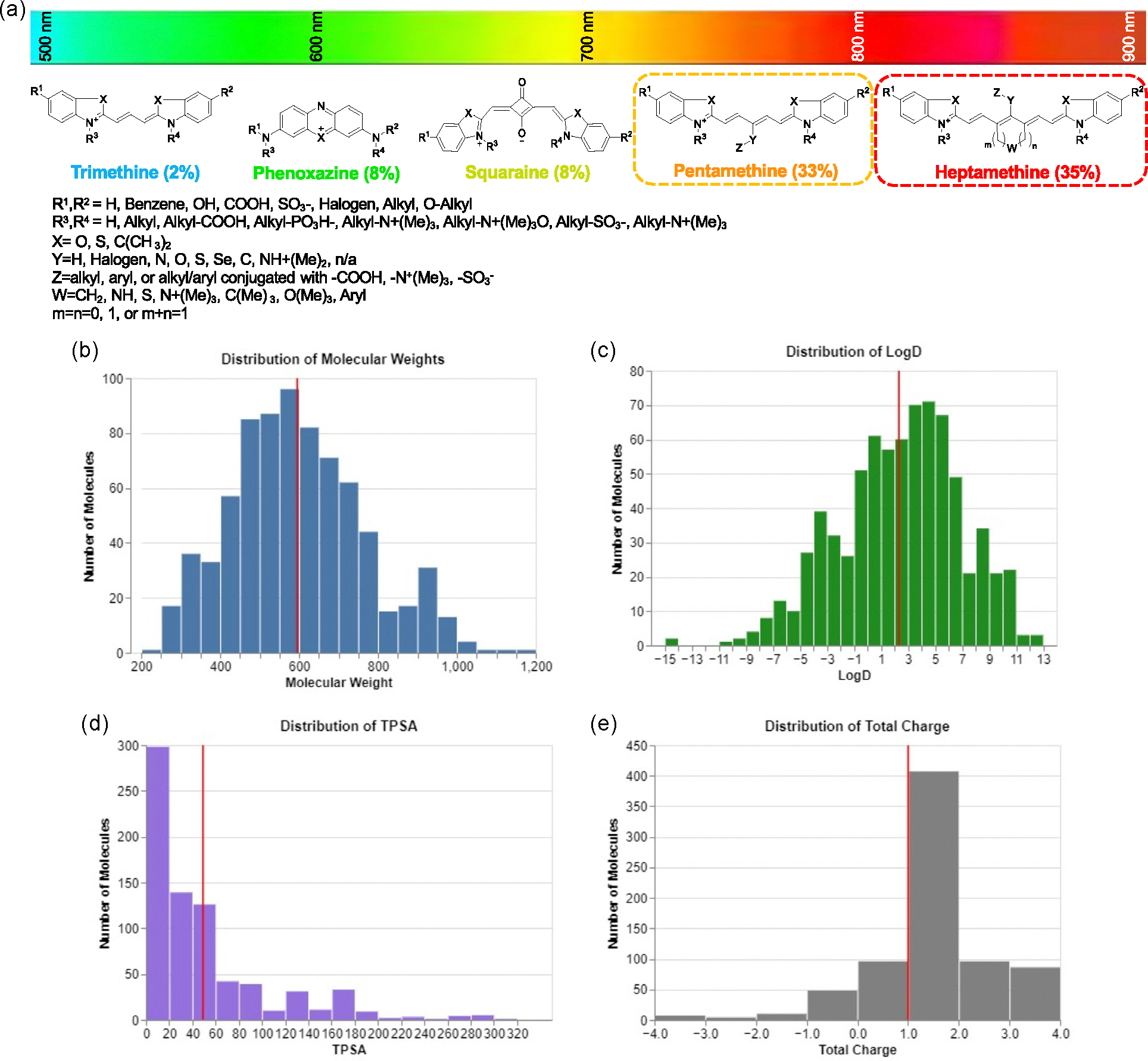
Structural diversity in the biodistribution database. a) The database contains molecules derived from substitutions on these common NIR fluorophores: trimethines, phenoxazines, squaraines, pentamethines, and heptamethines. Each skeleton’s respective makeup of the database is given as a percentage, with the most common skeletons, pentamethines and heptamethines, highlighted for further analysis in this study. Most molecules in the database are symmetric structures, where $R^{1}=R^{2}$ and $R^{3}=R^{4}$. These contrast agents cover the fluorescence at a wide spectrum from 500 nm (trimethines) to 800 nm (heptamethines). b–e) Each histogram shows the number of molecules within each range of MW given in Daltons (b), LogD at pH 7.4 (c), TPSA given in angstroms squared (d), and total charge (e), as displayed on each horizontal axis. The red vertical line of each histogram represents the average value of the database for the corresponding property.

The database contains the structure-inherent optical, physicochemical, and biodistribution properties of 764 contrast agents, with 250 pentamethines, 264 heptamethines, and 250 other fluorophores consisting of mostly phenoxazines, squaraines, and trimethines (Figure 1a). Even with a limited number of functional groups that may be substituents of or conjugated to the same structural skeletons, the combinatoric explosion creates a complex problem in the search for proper design of contrast agents, to the extent where this compound library is far from exhaustive. Therefore, finding ways to trim the bounding box of the search area is crucial for an efficient and productive design process for fluorophores.

In this exploration for physicochemical cutoffs lies the necessity for a diverse enough database that serves as a basis set for such a QSAR problem. In our database, a customized PowerPoint template is used to record each fluorophore’s properties, which are transferred to Instant JChem (Figure S1, Supporting Information). Some fluorophores were excluded from the current study due to their small sample size, namely BODIPY and rhodamine dyes. Phenoxazines and squaraines were also excluded from later analyses due to the same reason. For the structures with indole moieties, the most common substitutions were hydroxyls, halogens, sulfonates, and alkyl chains at the 5′ positions (R^1^ and R^2^), as well as substitutions on the indole nitrogen atoms (R^3^ and R^4^) with various alkyl chains ending with or without the quaternary ammonium, sulfonate, carboxyl, or benzene group. The 3′ position was normally demethylated carbon, oxygen, or sulfur atoms (X). It is worth noting that for pentamethines and heptamethines, substitutions with atoms including nitrogen, oxygen, or sulfur (Y) connected with different alkyl and aryl groups (Z) at the mesocarbon position of the polymethine chain were normal. A similar strategy of substitutions on the nitrogen atoms was applied when developing phenoxazines and squaraines. Due to the difference between the polymethine chain and other chemical skeletons, the fluorescence wavelength of our compound library covers a wide range, from visible to the NIR (500–850 nm).

As shown in Figure 1b, the fluorophores have an average MW of around 600 Da, with most fluorophores between 400 and 800 Da. Overall, the database shows broad logD (at pH 7.4) values from −15 to 12, being slightly more lipophilic with an average logD over 2.0 (Figure 1c). Because alkyl chain substitutions were the most common, the average TPSA in the database is relatively low, around 50 Å^2^, with almost half of the molecules in the database having a TPSA of less than 20 Å^2^ (Figure 1d). Interestingly, due to the inherent positively charged nitrogen in the indole ring or phenoxazine contained in most of the fluorophores, the average of the total charge is very close to 1 (Figure 1e). Regarding specifically the different structural skeletons, polymethines had similar spread in logD and TPSA, with pentamethines being slightly more hydrophilic overall (Figure S2, Supporting Information). Phenoxazines were clustered in a region of relatively low TPSA and slight hydrophobicity, and trimethines and squaraines have too few samples to see a clear pattern.

Identification of unavoidable patterns in the distribution of physicochemical properties within the database is essential for recognizing discovered trends and substantiating the diversity of the database. For example, as the skeletal structures of pentamethines and heptamethines have a MW of about 350 Da, the number of molecules greatly increases after the 400 Da threshold. Additionally, the indole rings seem to contribute to the overall lipophilicity, leading to a heavily right-skewed distribution of the TPSA and the positive charge. However, since these patterns are easily explainable, arise from fundamental properties of the fluorophore library, but do not ultimately limit the range of each property, we believe these trends do not challenge the diversity of the database.

### 2.2. Scoring Function Minimizes Loss of Data

**Figure 2.**
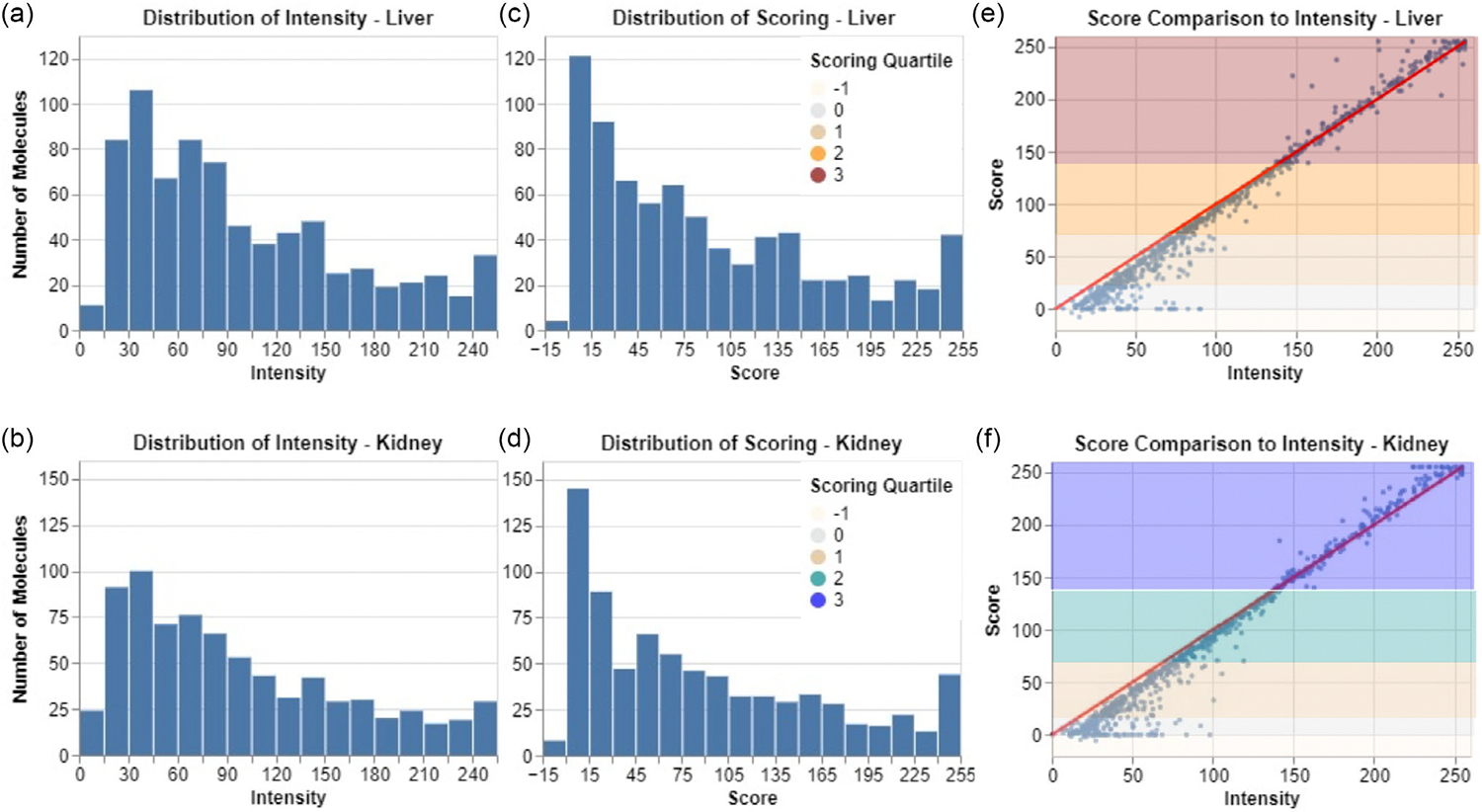
Distribution of raw intensity and scoring, comparison of metrics. a,b) The distribution of raw intensity measured from the biodistribution data for the a) liver and b) kidney is shown in a histogram. c,d) The distribution of the scoring function (Equation (1)) on the c) liver and d) kidney is shown in a histogram. e,f ) The scatterplot between the raw intensity and the score shows their relationship compared to the y=x line, in red, for the e) liver and f ) kidney. The colors of the scatterplot distinguish the quantile of the scores, where −1 represents negative scores and 0–3 represent quartiles that exclude the negative values.

Intravenously administered drugs typically distribute rapidly to the liver and kidneys during systemic circulation, followed by hepatobiliary and renal clearance, the major pathways involved in the removal of a xenobiotic.^[18–20]^ Therefore, we focused our attention on the signals accumulated in the liver and kidneys. All the biodistribution data were obtained 4 h postinjection intraoperatively; then the major organs were resected to create detailed scoring charts (Figure S1, Supporting Information). The signals in the liver and kidneys show relatively low intensities due to the systemic excretion of intravenously injected substances to the urinary bladder and feces (Figure 2a,b). Some of the low intensities could also be attributed to brighter system backgrounds due to the slight inconsistencies in the collected images during the past decade using different imaging systems. Therefore, attempts at standardized quantification of biodistribution data were made through conventional scoring algorithms, but they showed unsatisfactory distortion of the original distribution (see Figure S3 and S4, Supporting Information). The various shortcomings of these algorithms led to the development of Equation (1), whose distribution was similar to the original raw intensity, with greater density on the lower scores without heavily sacrificing the density in the higher scores (Figure 2c,d). Comparing the raw intensity with the score in a scatter plot (Figure 2e,f ) revealed that the equation does not favor one end of the distribution compared to the other, and any bias was an artifact of the original distribution of raw intensity, rather than a manufactured one resulting from the scoring process. Using colors to show the categorization into scoring quantiles, labeled with,1 to 3, also showed the same higher density in the lower scores, and points near y=0 of the scatter plots represented successful filtering of those molecules that had a high raw intensity in these organs due to long exposure time (high-system background intensity) and/or nonspecific distribution (high muscle intensity). The quantiles also allowed for a label threshold of 2 for high-scoring contrast agents, such that the chosen agents would have, approximately, the upper half of organ uptake. This would also ensure easier identification of discovered trends.

(1) $${Score}_{i}=c\left({ORG}_{i}\right)-c\left({MU}_{i}\right)$$

where

(2) $$c(x)=min\left(max\left(\frac{127.5}{127.5\;-\;BG}\times (x-127.5)+127.5,0\right),255\right)$$

Equation (1): Scoring Equation. The distribution score is the difference between the adjusted intensities of the organ $\left({ORG}_{i}\right)$ and muscle $\left({MU}_{i}\right)$ for fluorophore i, where the raw intensity x is adjusted with the exposure adjustment function (EAF, Equation (2)) c under the assumption that the system background (BG) is adjusted to be 0. The EAF uses the point-slope formula of a line through the midpoint of intensity (127.5, 127.5) and the background (BG,0) to map the intensity x to an adjusted intensity c(x). The minimum and the maximum functions exist to make sure the resulting value is within the range of 0 to 255. Then, c(x) is exactly the intensity of x after the entire image is contrast-adjusted such that the system background has a brightness of 0. This ensures proper standardization of the entire image based on information from the system background. The EAF is based on contrast adjustment in image processing software like ImageJ (version 1.52p).

### 2.3. Influence on Distribution to the Liver

**Figure 3.**
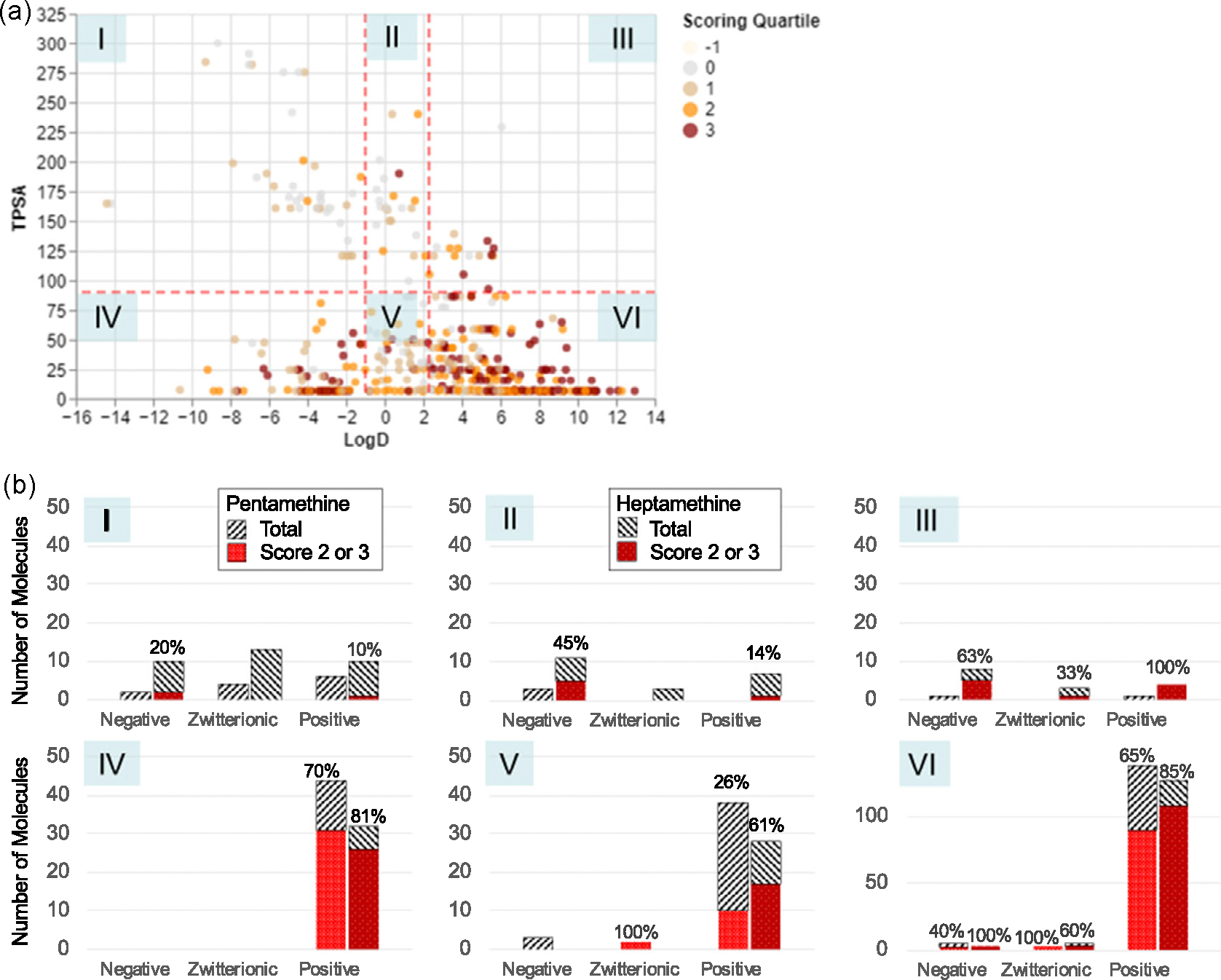
Effect of physicochemical properties on liver uptake. a) Each point in the scatter plot is colored by the scoring quantile of the molecule, as per Figure 2. The visual clustering in two regions of the chemical space is segmented off by the dotted red lines at LogD = −1, 2.3 and TPSA = 90 Å^2^. b) The bar charts correspond to the spaces denoted with the roman numerals. Each bar chart also shows the percentage of the pentamethine and heptamethine fluorophores, with the corresponding total charges denoted on the horizontal axis, which has high distribution to the liver (with scores of 2 and 3). Note the different y-axis scale of the bar chart for region VI, due to the varied data density.

As a majority of the compounds in the database are pentamethines and heptamethines (67%), we focused on these skeletons for biodistribution analysis. For the distribution to the liver, 314 (61%) compounds are found to be in scoring quantiles of 2 and 3, while there are 138 pentamethines (55%) and 176 heptamethines (67%). Figure 3a shows a greater visual density of highly distributing molecules in regions IV (76 compounds) and VI (281 compounds), where TPSA is less than 90 Å^2^ and either logD is less than −1 or greater than 2.3, respectively. The choice of these thresholds was guided by the depiction in the scatterplot. Interestingly, region IV had the highest significant distribution with pentamethines, with 70% of the 44 positively charged pentamethines and 81% of the 32 heptamethines having a high distribution score, where most have +2 or +3 charges (Figure 3b and S5a, Supporting Information). On the other hand, region VI has the highest significant distribution with heptamethines, with 85% of the 127 positively charged heptamethines and 65% of the 138 pentamethines having a high distribution score, where most have +1 charge (Figure S5a, Supporting Information). Other regions also showed some distribution to the liver, but these were out of smaller sample sizes, with usually less than ten molecules in those categories. Overall, lipophilic cations and some hydrophilic cations with low polar surface area tended to end up in the liver.

### 2.4. Influence on Distribution to the Kidney

**Figure 4.**
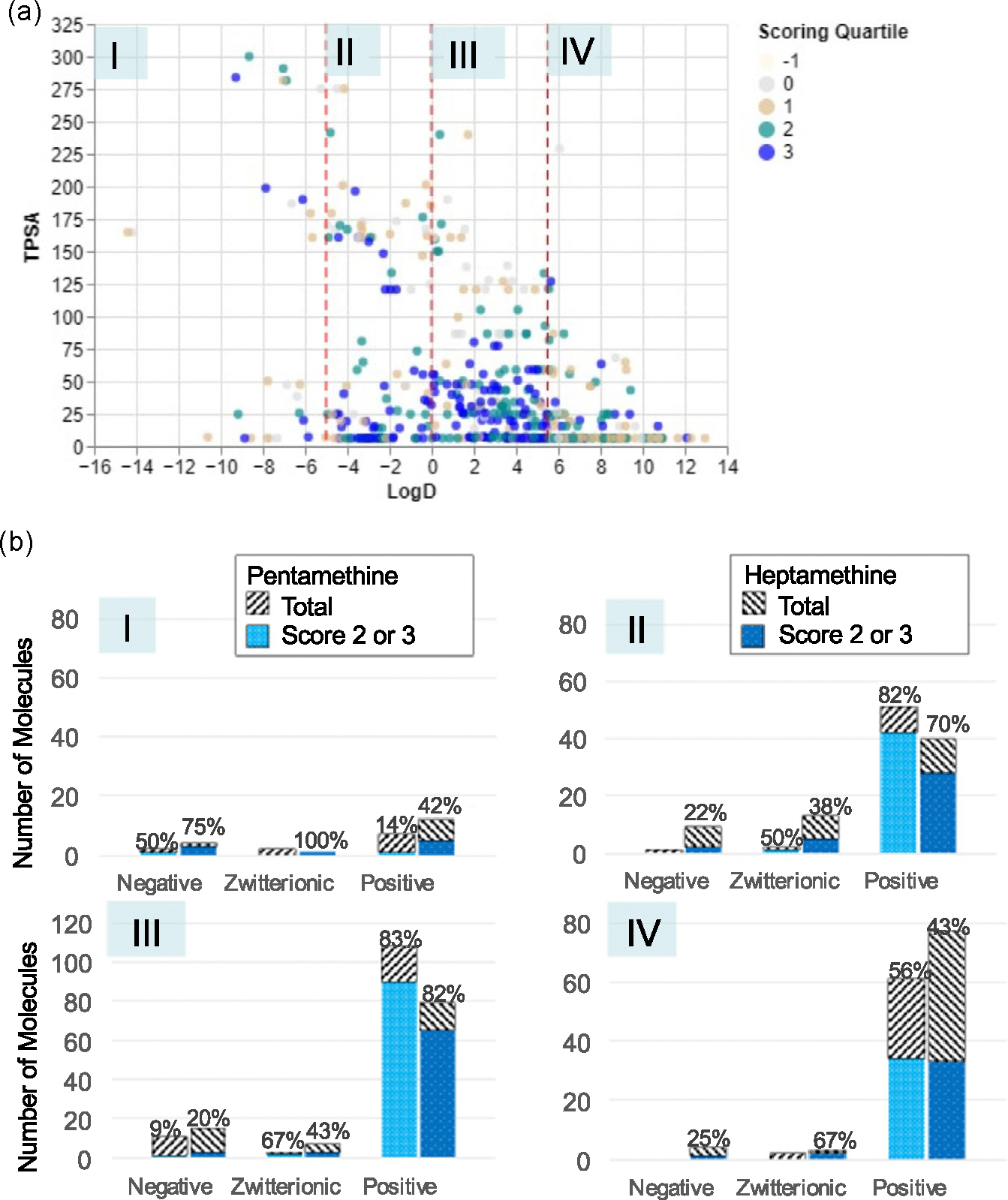
Effect of physicochemical properties on kidney uptake. a) Each point in the scatterplot is colored by the scoring quantile of the molecule, as per Figure 2. The visual clustering in the chemical space is segmented off by the dotted red lines at LogD = −5, 0, and 5.5. b) The bar charts correspond to the spaces denoted with the roman numerals. Each bar chart also shows the percentage of the pentamethine and heptamethine dyes, with the corresponding total charge denoted on the horizontal axis, which has high distribution to the kidney (with scores of 2 and 3). Note the different *y*-axis scale of the bar chart for region III, due to the varied data density.

Out of the 514 pentamethines and heptamethines, 323 (63%) are found to be in scoring quantiles of 2 and 3 in kidney uptake, whereas there are 172 pentamethines (69%) and 151 heptamethines (57%). Figure 4a shows the visual density of those with high-scoring distributions in regions II and III, where logD is between −5, 0, and 5.5. These thresholds were chosen from the visual representation in the scatterplot. TPSA-guided division was not used due to the significant existence of high-scoring molecules with TPSA > 100 Å^2^. In region II, 82% out of 51 positively charged pentamethines and 70% out of 40 positively charged heptamethines have a high distribution score, while most have +2 or +3 charges (Figure 4b and S5b, Supporting Information). Similarly, in region III, 83% out of 108 positively charged pentamethines and 82% out of 79 positively charged heptamethines showed high distribution scores, whereas most have a +1 charge. Like Figure 3, other regions also showed some distribution to the kidney, but these were out of smaller sample sizes, usually less than 10. Overall, cations with near-zero logD tended to end up at the kidney.

### 2.5. Statistical Significance of the Segmentation of Chemical Space

Table 1 summarizes the probability of achieving these results from random selection. The table lists the number of total and high-scoring contrast agents of a certain type (y, x, respectively) and the number of total and high-scoring agents of that type within the region in question (n, m, respectively). For example, in row 1, there are y=250 pentamethines in total, x=138 of which are high-scoring, n=44 of which are in region IV, and m=31 of which are both high-scoring and in region IV. The probability p that there are n high-scoring fluorophores out of m random samples, without replacement, from y total fluorophores with x high-scoring fluorophores is calculated by the hypergeometric distribution probability mass function. As this measures the probability arising from random selection, it is equivalent to the probability that the selection itself was random, thus supporting the statistical significance of these choices of chemical space segmentation.

For the liver, the probability that 70% of 44 randomly selected positively charged pentamethines are high scoring was found to be 0.01, and the probability that 85% of 127 randomly selected positively charged heptamethines are high scoring was less than 5 × 10^−10^. For the kidney, the probability this distribution would occur from a random sampling of our database was less than 1.0 × 10^−5^ for pentamethines and less than 1.0 × 10^−9^ for heptamethines. Additionally, the total probability that m would be any value greater or equal to the values in the table also resulted in *p* < 0.02, further supporting the significance of the choice of the thresholds.

### 2.6. Polar Descriptors

Besides the TPSA and LogD, other properties such as the net charge, number of rotatable bonds, and number of hydrogen bonds showed close relationships with renal and hepatobiliary clearance (Figure S6, Supporting Information).^[18–21]^ In general, the influence pattern of polar descriptors on hepatobiliary clearance is less clear due to the limited number of fluorophores getting stuck in the liver, with the largest percentage stuck being zwitterionic (Figure S6a,b, Supporting Information). On the other hand, a significant number of fluorophores with a +1 or +2 charge were stuck in the kidney, whereas zwitterionic fluorophores (net charge of 0) had a smaller percentage stuck in the kidneys (Figure S6a, Supporting Information). Interestingly, for the 261 pentamethines and heptamethines that carry only one quaternary ammonium on the indole nitrogen (+1 net charge), 157 (60%) were stuck in the kidney. Generally, the patterns of rotatable bonds and hydrogen bonds were similar, where the increase of rotatable or hydrogen bonds in pentamethines and heptamethines led to decreased hepatobiliary clearability (Figure S6b, Supporting Information) but increased renal clearance (Figure S6c, Supporting Information).

In general, renal clearable compounds tend to show higher TPSA, number of rotatable bonds and hydrogen bond count, and hydrophilicity (more negative logD values). For fluorophores in region II of Figure 4, increasing the positive charges seemed to improve the ratios of pentamethines and heptamethines that were successfully distributed to the kidney. For fluorophores in region III, similar patterns did not show, possibly due to the smaller number of +2 and +3 charged fluorophores. LogD values in this region are also normally higher (0–5.5), which may negatively impact fluorophore’s systemic clearance. Additionally, renal clearance may relate to the compensations of high hydrogen bond count and rotatable bonds (Figure S6c, Supporting Information).

### 2.7. Multiple Properties Simultaneously Govern Distribution and Excretion

**Figure 5.**
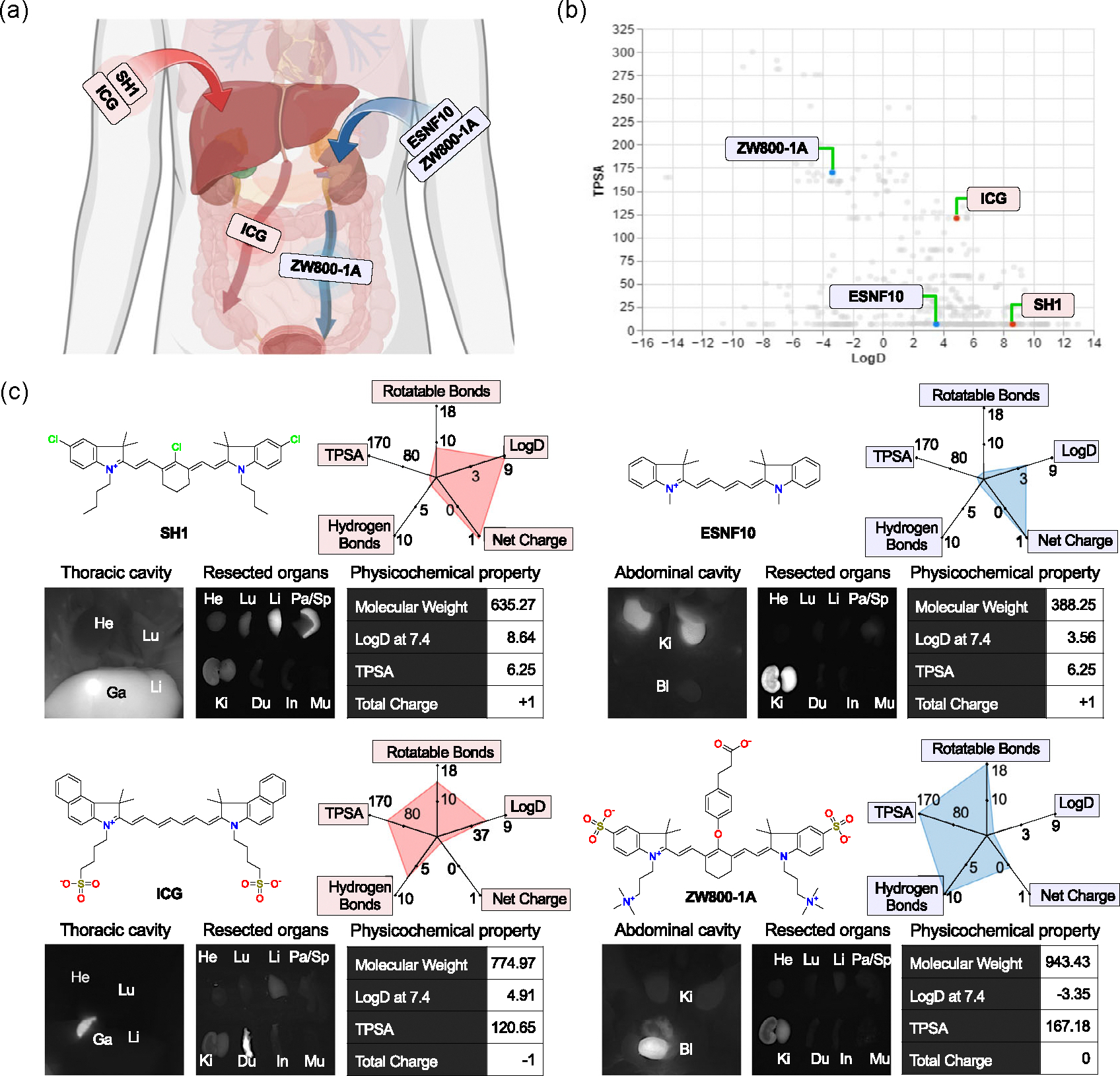
Representative examples of other physicochemical properties impacting distribution and excretion. a) Major uptake and clearance routes of four molecules of the database, where SH1 is mostly stuck in the liver and ICG is cleared through the liver, while ESNF10 is mostly stuck in the kidney and ZW800–1 A is cleared through the kidney. b) The logD versus TPSA scatterplot, as seen in Figure 3 and 4, shows the location of these fluorophores in this chemical space. c) The molecular structure, table and radar chart of physicochemical properties, and NIR cavity/organ images of each molecule are shown. Each radar plot graphs extended physicochemical properties of each molecule, with minimum and maximum for each property chosen from the four molecules.

Figure 5 shows representative examples of hepatobiliary and renal clearable NIR fluorophores. Among them, indocyanine green (ICG) is an FDA-approved 800 nm fluorophore with a LogD of 4.91 and TPSA of 120.65 Å^2^ (region III). Due to the short blood half life of ICG (*t*_1/2_: 2–4 min), the signal intensity in the liver was low compared to the high intensity in the duodenum.^[22]^ In comparison, SH1 (*t*_1/2_: 5.39 ± 1.53 h) is a tumor-targeted 800 nm fluorophore that showed high uptake in the liver, gallbladder, and spleen, but did not complete excretion through the hepatobiliary route by 4 h, possibly due to its relatively higher lipophilicity and low TPSA (Figure 5c).^[23]^ For ZW800–1 A, the zwitterionic property, low LogD value (−3.35), high TPSA (167.18 Å^2^), and high flexibility (18 rotatable bonds) may have governed its fast and high renal clearance (*t*_1/2_: 15 min) with minimum uptake in the liver and other organs.^[13,22]^ As for endocrine-tissue targeted ESNF10, it showed high signal intensity in the kidney which may be due to the lower TPSA (6.25 Å^2^), rotatable bonds, and hydrogen bonds.^[24]^

### 2.8. Other Influencing Factors

Aqueous solubility does play a determining role in the biodistribution and excretion of drugs. Recent studies have shown the advantages of ionized or zwitterionic forms for solubility enhancement compared to the solubility of neutral molecules.^[25,26]^ To that end, the number of substitution groups such as COOH, SO_3_^−^, and N(CH_3_)_3_^+^ on the skeleton are normally used to adjust the net charge and solubility. Unfortunately, due to the number of synthesized fluorophores, the real solubilities in aqueous solvents were difficult to measure, and thus logD at pH 7.4, calculated by MarvinSketch, was used in the analysis. Therefore, it is important to note that there may be a bias in these calculated values, especially for compounds carrying different numbers of quaternary ammoniums.

Due to the variety of the physicochemical properties of the fluorophores and the complexity of in vivo environments, the blood half-life time of each fluorophore varies. However, to simplify the process of biodistribution study of the designed fluorophores, we standardized the working solution for the biodistribution evaluations and imaging time point at 4 h post-injection. Therefore, there is a chance to get a high duodenal or bladder signal but low liver or kidney signal for compounds with faster hepatobiliary or renal clearance rate (leading to lower scoring quartile occupancy in Figure 3 and 4), or a high signal in the liver or kidney for compounds getting stuck due to the slower clearance rate, solubility, charge, or interactions with related transporters.

Despite this, some molecules can be seen to be highly-distributing but not in a highlighted chemical region (such as Region VI in Figure 3a), or vice versa. These can be due to various factors, from engineered functional groups to biochemical mechanisms affecting distribution. Note that a significant number of molecules in the compound library was formulated with a specific biodistribution, clearance, or targeting tissues or diseases and thus may not follow the discovered trend. Moreover, as physicochemical properties are not the only factors that affect biodistribution, other mechanisms, such as serum protein or glycoprotein binding, may have an impact greater than the patterns that physicochemical properties alone can reveal.^[15,27]^ However, these biochemical processes are beyond the scope of this study, in which we focus on the general trend found in the overall properties of small molecules.

Further application of this compound library is promising. This scoring method can continue to be applied to expansions on the database for classification or regression analysis using machine learning (ML) methods, which may provide further insights into the nature of biodistribution. Quick implementation of common ML algorithms showed the database’s potential for training and effective prediction (Figure S7, Supporting Information). ML applications could even lead to the prediction of large molecular libraries created through rote combinatorics, the interpretation of the weight of impact for specific functional groups on the biodistribution of a molecule, or the generation of novel compounds through algorithms like generative adversarial networks.^[28]^ Not only this, but we can also expand this analysis on this database further from the liver and the kidney to other organs and even specific tissues such as nerves or lymph nodes for disease-specific contrast agent design.

## 3. Conclusion

In summary, the effectiveness of NIR fluorophores for targeted imaging heavily depends on the customizability of the fluorophores themselves. As such, improving the efficiency of designing and developing these contrast agents is essential for continued progress in the field of medical NIR imaging. To aid in this process, we have developed a biodistribution database with a large and diverse compound library that can be used as a basis for further fluorescent contrast agent design. In analyzing this database, we have found that certain physicochemical properties, such as polar descriptors, lipophilicity, and ionization state, affect biodistribution and uptake to certain organs, specifically the liver and kidney. These physicochemical properties can be easily altered in a fluorophore to manipulate the disposition and achieve a pharmacokinetic advantage. We aim to continue to refine this strategy to aid in future NIR contrast agent design for specified biodistribution and fluorescent imaging.

## 4. Experimental Section

### NIR Fluorescence Imaging System:

For dual-channel NIR imaging, 650 nm excitation light (700 nm NIR channel) at 4.0 mW cm^−2^ and 760 nm excitation light (800 nm NIR channel) at 1.0 mW cm^−2^ were used with white light (400–650 nm) at 5,500 lux. Simultaneous color images (512 × 512 pixels) with the choice of either 700 or 800 nm fluorescence images were acquired using an AD-130GE camera (JAI, Yokohama, Japan) installed with a custom-dual bandpass prism (700 nm channel: 710/50, bandpass filter, 800 nm channel: 780 longpass filter). The imaging system was controlled by custom software at rates up to 15 Hz, except for the field of view which was manually adjusted by macrozoom lens (0–10X; Navitar Zoom 7000). The imaging head was positioned 9–18 in. from the surgical field, and all NIR fluorescent images had identical exposure times and normalizations.

### NIR Imaging in Mice:

To standardize the biodistribution data, we injected 10–25 nmol of each fluorophore intravenously into *n* = 3 CD-1 mice. The in vivo behavior of the fluorophores was carefully recorded after 4 h of injection. An optical and NIR image of each region of the mouse was recorded, after which the organs were placed on a black system background and recorded for a biodistribution image. The images of the regions and the biodistribution were then translated into our database, created with Instant JChem (ChemAxon, Budapest, Hungary).

### Preparation of the Biodistribution Database:

For each entry in the Instant JChem database, each biodistribution image was analyzed using ImageJ (version 1.52p). From each image, we recorded the average value, or “intensity,” of the pixels contained in each organ, the muscle, and the system background, to a number between 0 and 255. The database was purged of duplicate or incomplete entries, and entries that were found to be toxic or led to mouse death from injection were omitted.

### Analysis of the Biodistribution Database:

The average intensity of the organs and the physicochemical properties of each molecule were compiled with a python notebook (Google Colab). The numpy and pandas libraries were mainly used for data organization and analysis. As each dye fluoresced with varying brightness, the raw average intensity was unable to provide a clear comparison between the molecules and their ability to distribute to different organs. Therefore, each intensity of the organs was manipulated via Equation (1) to standardize each intensity and provide a reliable distribution scoring method.^[29]^ From this scoring, each molecule was placed into a scoring quantile, where −1 represented a negative score, and 0–3 represented the quartiles of the remaining molecules in ascending order. Data visualization of some of the figures was created with the altair library. Due to the volume of the data and for clarity, only pentamethines and heptamethine fluorophores were considered in the scatterplot and bar charts.

## Supplementary Material

Supplementary Information

## Figures and Tables

**Table 1.** Probability assuming a random distribution of fluorophores in high-scoring quantiles.

Biodistribution in Regions	*x* ^a)^	*y* ^b)^	*m* ^c)^	*n* ^d)^	*p*

Liver, Pentamethines, Region IV	138	250	31	44	0.0107
Liver, Heptamethines, Region VI	176	264	108	127	4.46 × 10^−10^
Kidney, Pentamethines, Region III	172	250	90	108	7.10 × 10^−6^
Kidney, Heptamethines, Region III	154	264	65	79	8.46 × 10^−8^

a) *x*: the total number of high-scoring agents of the given type (column 1).

b) *y*: the total number of that type of contrast agent.

c) *m*: the number of high-scoring agents of that type in each region.

d) *n*: the number of agents of that type in each region.

## Data Availability

The data that support the findings of this study are available in the supplementary material of this article.
