# Supplementary material for: Physicochemical Descriptors in Biodistribution and Clearance of Contrast Agents
Source: Adv Photonics Res. Author manuscript; Available in PMC 2025 Aug 26. (PMC12377636; doi:10.1002/adpr.202300036)
Supplement: Supplementary Information [file NIHMS2054599-supplement-Supplementary_Information.pdf]

Supporting Information

Physicochemical Descriptors in Biodistribution and Clearance of Contrast Agents

Sung Ahn,[1] Benjamin Sanchez-Langeling,[2] Jeong Heon Lee,[1] Maged Henary,[3*] Kai Bao,[1*] and Hak Soo Choi[1*]

[1]Gordon Center for Medical Imaging, Department of Radiology, Massachusetts General Hospital and Harvard Medical School, Boston, MA 02114, USA

[2]Department of Chemistry and Chemical Biology, Harvard University, Cambridge, Massachusetts 01238, USA

[3]Department of Chemistry and Center of Diagnostics and Therapeutics, Georgia State University, Atlanta, GA 30303, USA

*Correspondence to K.B. kbao@mgh.harvard.edu; M.H. mhenary1@gsu.edu; H.S.C. hchoi12@mgh.harvard.edu

The PDF file includes:

Supplementary Methods

Figure S1. Representative database entry for ICG.

Figure S2. Distribution of structural backbones in physicochemical space.

Figure S3. Distribution of Scoring Method and Comparison to Raw Intensity.

Figure S4. Distribution of System-Corrected Signal-Muscle Ratio and Comparison to Raw Intensity.

Figure S5. Effect of Positive Charges on Kidney Uptake.

Figure S6. Effect of Polar Descriptors in Clearance.

Figure S7. Machine Learning Results.

SUPPLEMENTARY METHODS

Scoring Calculations and Machine Learning (ML) Algorithms: All calculations and algorithm implementations were done on a Google Colab notebook running Python 3. Data processing was done using the numpy[1] and pandas[2] libraries. Data visualization utilized the altair [3] library. In the following equations, x stands for organ intensity, y stands for the muscle intensity, z stands for the system background intensity, and b stands for background, which can be the muscle or the system background intensity.

ML algorithms were processed on the pentamethine and heptamethine slices of the database with algorithms available from the sklearn [4] library and the results were visualized with the seaborn [5] library. They were trained to learn different subsets of physicochemical properties (logD, molecular weight, strongest acidic/basic pKa, TPSA, chirality, hydrogen bond acceptors/donors, rotatable bonds, total charge, LogP) on varying slices of included structures (pentamethine/heptamethine only, or both included) for four different target classification labels: the liver/kidney uptake score quantile (LIQ/KIQ) and the hepatobiliary/renal clearance (HC/RC). The different subsets of physicochemical properties are as follows: 'all' includes all properties; 'select' includes logD, molecular weight, TPSA, hydrogen bond information, rotatable bonds, and total charge; 'altselect' includes those in 'select' without molecular weight and logD replaced with logP; 'altselect2' includes those in 'select' without molecular weight and logP included; 'polar' includes TPSA, logD, total charge, rotatable bonds, and hydrogen bond information; 'npolar' includes the normalized 'polar' information based on each property's range in the database.

The ML algorithms were selected based on availability and applicability to the database. As presented in **Figure S7a**, the chosen algorithms were random forest, k-nearest neighbors, gaussian process classifier, decision tree classifier, AdaBoost[6] classifier, and the gaussian naïve bayes classifier. After iterating $n = 3$ times on each feature (6), target (4), and structure inclusion (3), a box plot of the 216 results for each model showed better scores for the random forest algorithm overall (**Fig. S7a**). A similar method was used for comparison of the predictability of each target using the random forest algorithm, with each model iterated $n = 15$ times for a total of 45 results for each target-feature pair (**Fig. S7b**).

Contrast: $$y_C = x - b \qquad \text{(Eq. S1)}$$

The contrast equation finds the strict difference between the intensity of the organ in question with a background. However, this algorithm is unable to consider multiple reference points, as well as maintain its potency with extreme values of intensities. For example, a less significant result of 80 intensity with background 100 has the same contrast with a 20 intensity with background 0; similarly, a 180 intensity with background 200 results in the same contrast as well. Not only this, overall, the contrast can only reduce the score from the raw intensity, and never improve upon it as a reward for darker system background and muscle intensity (**Fig. S1**). Therefore, the score distribution was mostly a shift of the raw score distribution towards the negative direction.

Adjusted Weber Contrast: $$y_W = 255 * \frac{x-b}{x} \qquad \text{(Eq. S2)}$$

The original Weber Contrast [4] finds the ratio of the contrast between the intensity of the organ and the raw intensity of the organ, which results in a rational number between 0 and 1. The adjustment multiplies this ratio by 255 for easier comparison with the raw intensity. Using a ratio for comparison of signals uses a comparison which is more visual sensitivity-focused, due to the way the human brain compares visual signals.[7] This solves the problem of the reduction of potency with extreme values mentioned above, but this method of scoring heavily biases the higher extremes, making it difficult to spot more minute nuances between contrast agents (**Fig. S2**). Additionally, it creates extremely negative outliers due to certain fluorophores' biodistribution data having a brighter background with dimmer organs. Finally, this algorithm is also unable to consider multiple reference points.

Signal-to-Background Ratio: $$y_{SBR} = \frac{x}{b+1} \qquad \text{(Eq. S3)}$$

The signal-background ratio (SBR)[8] finds the ratio between the intensity of the organ with a background and thus uses similar visual sensitivity-oriented comparison as the Weber contrast. This method of scoring is used often in single fluorophore biodistribution analysis for comparison between the organs (**Fig. S1**, top-right). However, the signal-background ratio focuses on the brightness of the organ against the background, rather than contrast against the background. This led to extremely positive outliers, due to many background intensities near 0. Therefore, the divisor was adjusted such that the score would be closer to the raw organ intensity as the background got

darker. However, this still resulted in a heavy lower end bias, again creating difficulties in spotting nuances (**Fig. S3**). This algorithm also was unable to consider multiple reference points.

Contrast-to-Background Ratio: $y_{CBR} = \frac{x-b}{b+1}$ (Eq. S4)

The contrast-background ratio (CBR), similar to the signal-background ratio, finds the ratio between the contrast, instead of the raw signal of the organ, and a background. This method of scoring is also used often in single fluorophore biodistribution analysis. However, this is mathematically equivalent to $y_{SBR} + (\frac{1}{b+1} - 1)$, where the second term is between 0 and -1, and thus negligible. This led to a very similar score distribution as the SBR with much of the same inherent problems (**Fig. S4**).

System-Corrected Signal-Muscle Ratio: $y_{SCR} = \frac{x-z}{y+1-z}$ (Eq. S5)

The system-corrected signal-muscle ratio (SCR) attempted to fix the problems inherent in the previous scoring functions by naively correcting the values of the organ intensity and muscle intensity with the system background intensity, accounting for the varying exposure lengths. However, this led to more extreme outliers than the previous scoring methods, possibly due to fluorophores with just enough difference between the muscle and system background intensity, which led to a near-zero denominator (**Fig. S5**). Further inquiry into using the system background information for the adjustment of organ and muscle intensities led to using a contrast correction system based on image processing software, which in turn led to the development of **Equation 1**.

Operator	SN	Animal	CD-1	Chemical	ICG	Target Tissue	Ga	Sample prep date	4/8/19
Date	4/8/19	Body Weight	25-30 g	Working solution	250 μM	BioD Tissues	Du	Imaging Time	4 h
Model	K-FLARE	Gender	Female	Injection volume	100 μL	Clearance	HB	Exposure Time (ms)	100,200,400
Channel	800-nm	Inj. Route	R.O.	Formulation	10% BSA	Comments			

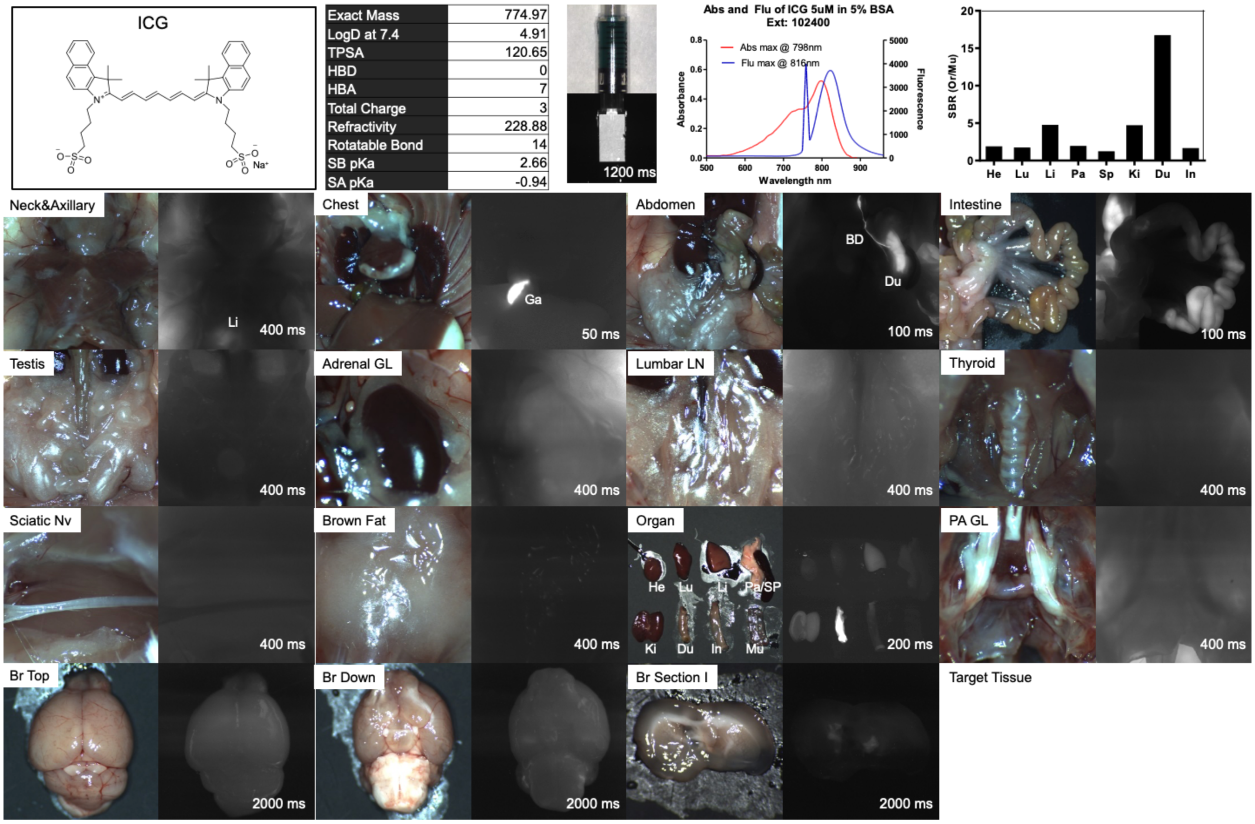

Figure S1. Representative database entry for ICG. The compound library is built on a standardized format, making note of the contrast agent's physicochemical, optical, and biodistribution properties. The target tissue, biodistribution, and clearance route as of 4 h post-injection are noted. Abbreviations used are: Bl, bladder; BD, bile duct; Du, duodenum; Ga, gallbladder; He, Heart; In, intestine; Ki, kidneys; Li, liver; Lu, lungs; Mu, muscle; Pa, pancreas; Sp, spleen. As a method for futureproofing, the model of the NIR fluorescence imaging system, the channel used, and information regarding the animal model and the injection are recorded, with room for further comments. Each NIR image is taken with an optical image for visual comparison, and the exposure times for each NIR image are recorded in milliseconds (ms). The datapoint is built on PowerPoint, after which the relevant information, including the optical properties and organ intensities as measured by ImageJ, are transferred to Instant JChem.

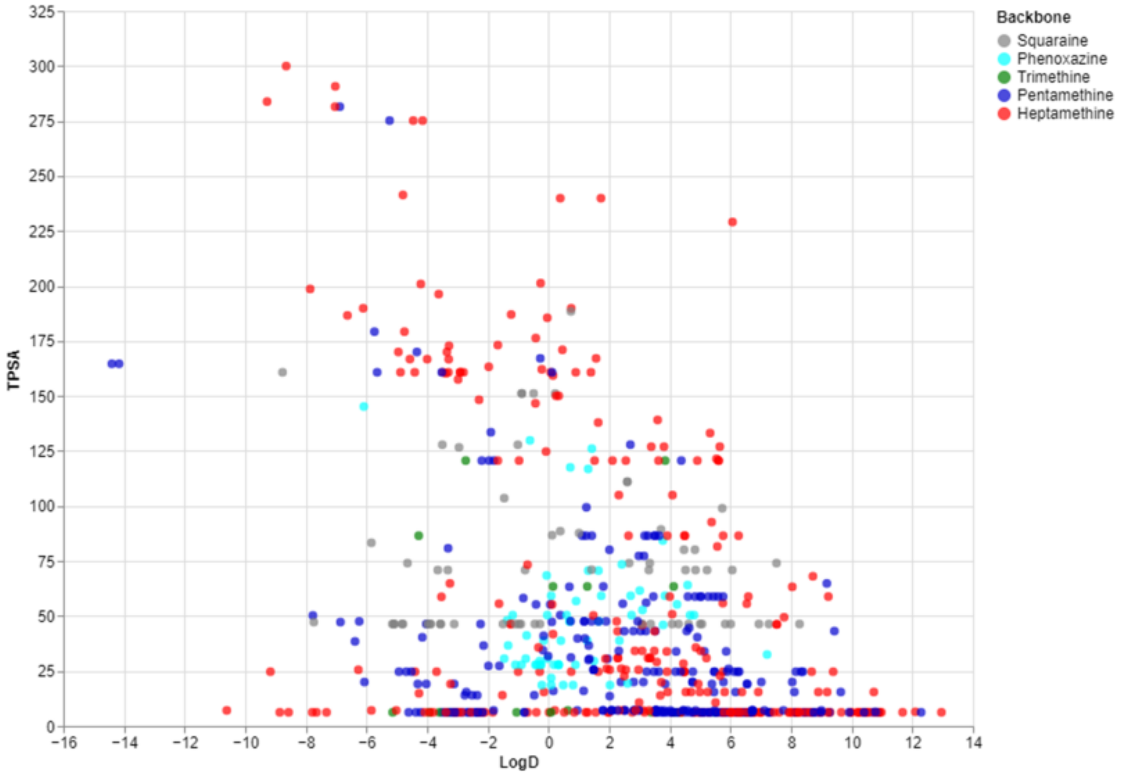

Figure S2. Distribution of structural backbones in physicochemical space. The compound library, mapped on the scatter plot by its physicochemical properties, LogD and TPSA, is colored by its structural backbones.

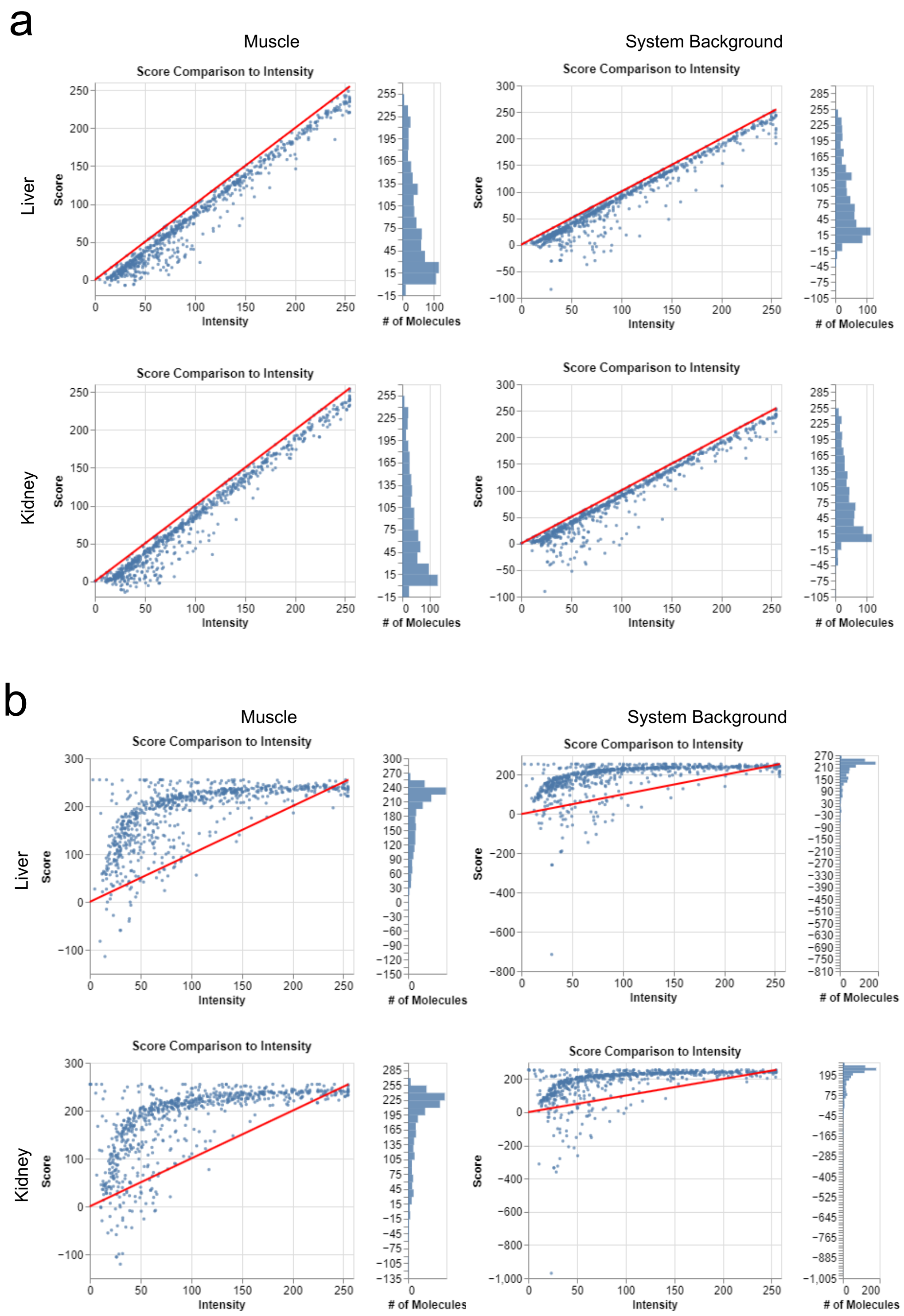
a
Muscle
System Background
Liver
Kidney
Score Comparison to Intensity
Score
Intensity
# of Molecules
b
Muscle
System Background
Liver
Kidney
Score Comparison to Intensity
Score
Intensity
# of Molecules

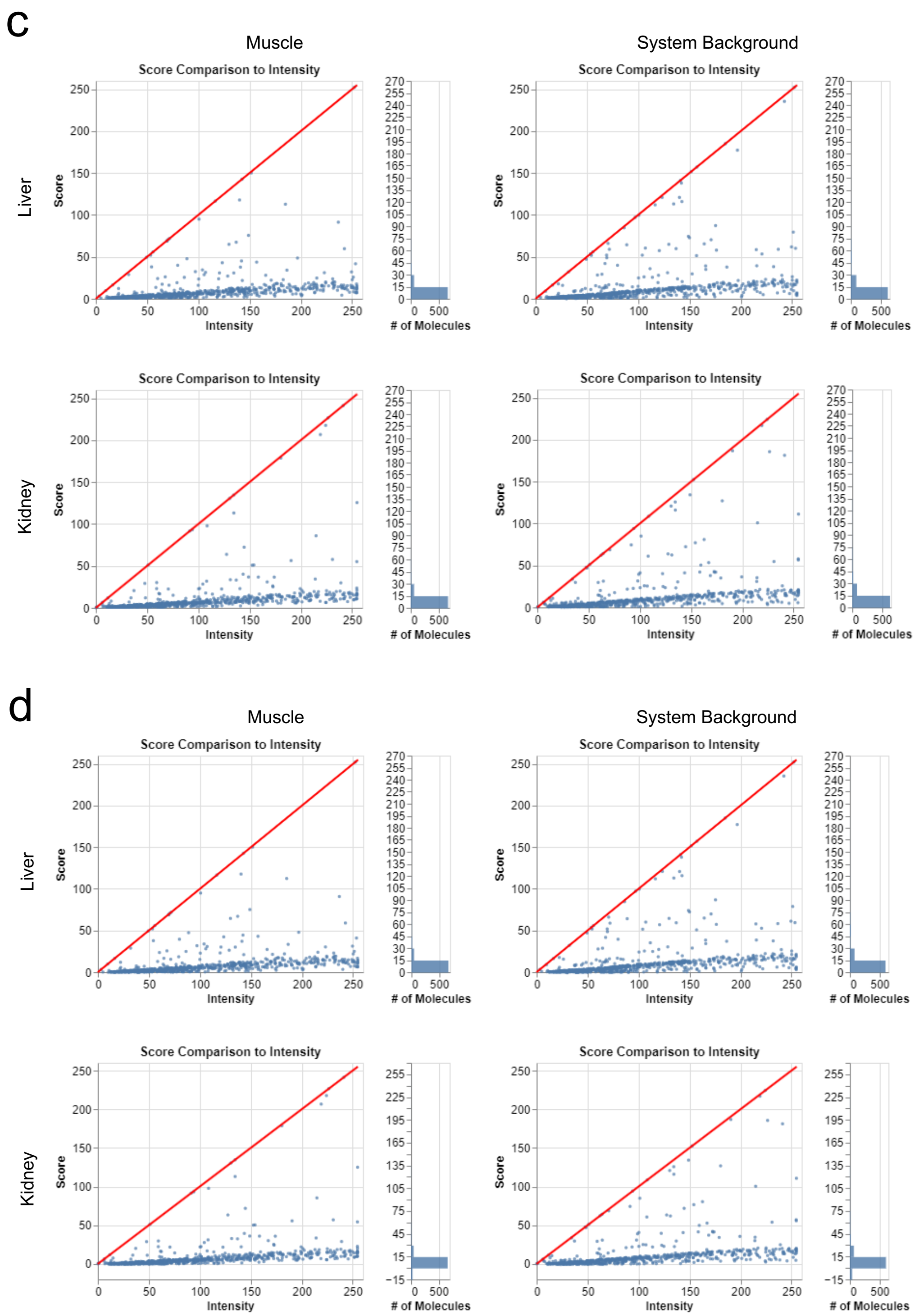

Figure S3. **Distribution of Scoring Method and Comparison to Raw Intensity** for the liver and kidney using either the muscle or the system background as a basis. Each red line depicts the $y = x$ line, where the score is equal to the raw intensity. The bar chart to the right of each scatter plot depicts the distribution of the data after the scoring function, where the function is based on contrast (a), Weber Contrast (b), Signal-Background Ratio (c), and Contrast-Background Ratio (d).

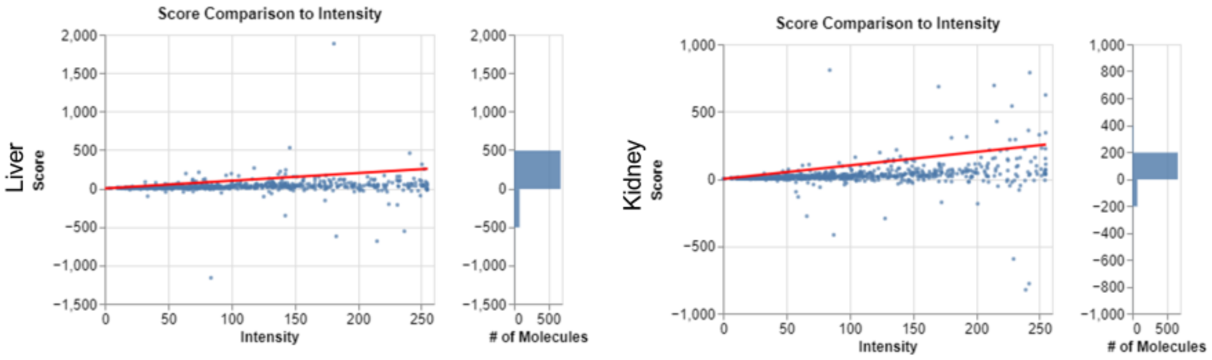

Figure S4. **Distribution of System-Corrected Signal-Muscle Ratio and Comparison to Raw Intensity** for the liver (left) and kidney (right). The red line depicts the $y = x$ line, where the score is equal to the raw intensity. The bar chart to the right of the scatter plot depicts the distribution of the data after the scoring function.

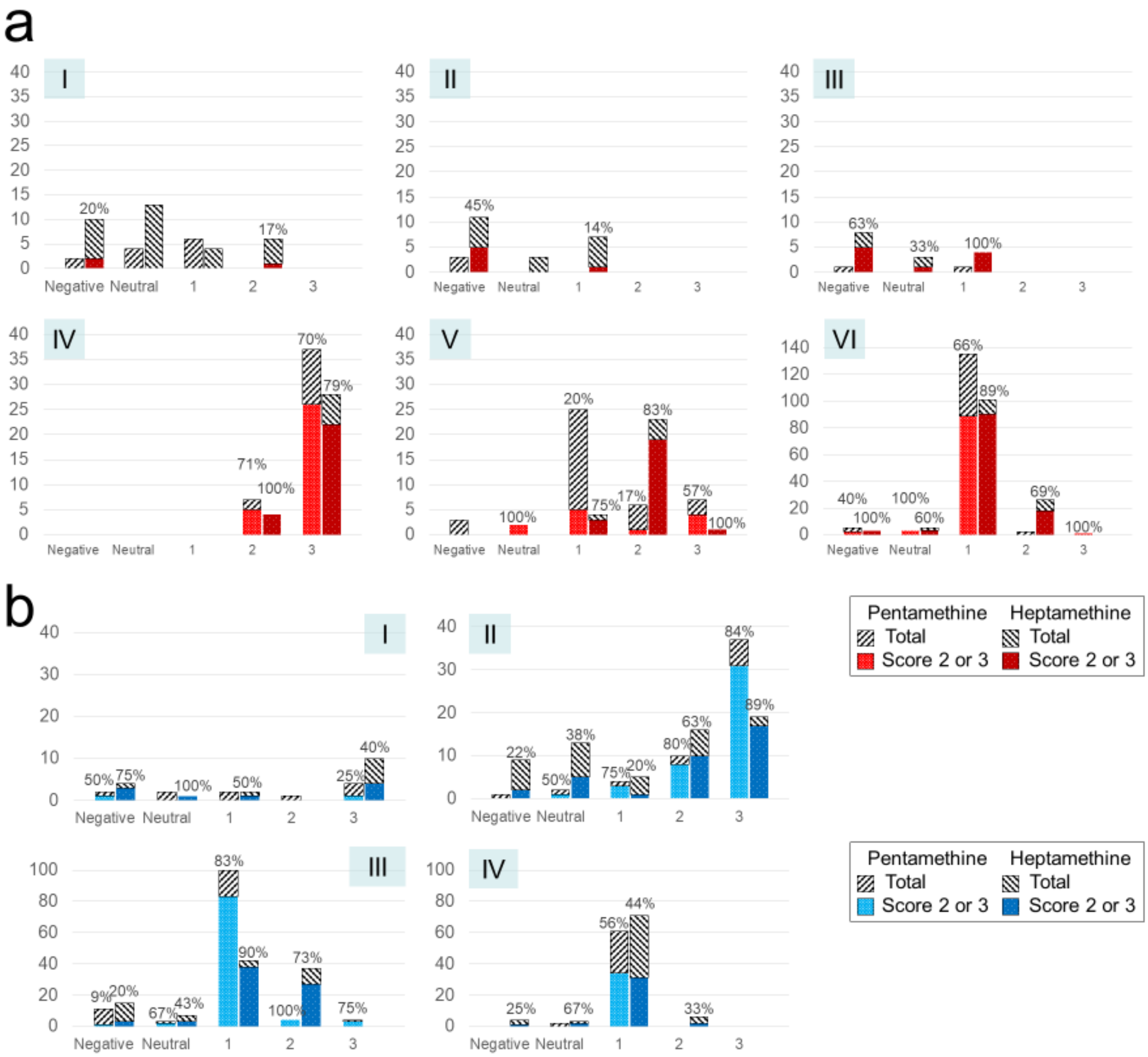

Figure S5. Effect of charge on liver and kidney uptake. The bar charts of the four regions from **Fig. 3-4** are extended to show the detailed liver (a) and kidney (b) uptake for different degrees of charged molecules. The y-axis denotes the number of molecules in each category. Each bar chart correlates with the corresponding scatter plots, with regions I-VI of the top set from **Fig. 3a** and regions I-IV of the bottom set from **Fig. 4a**.

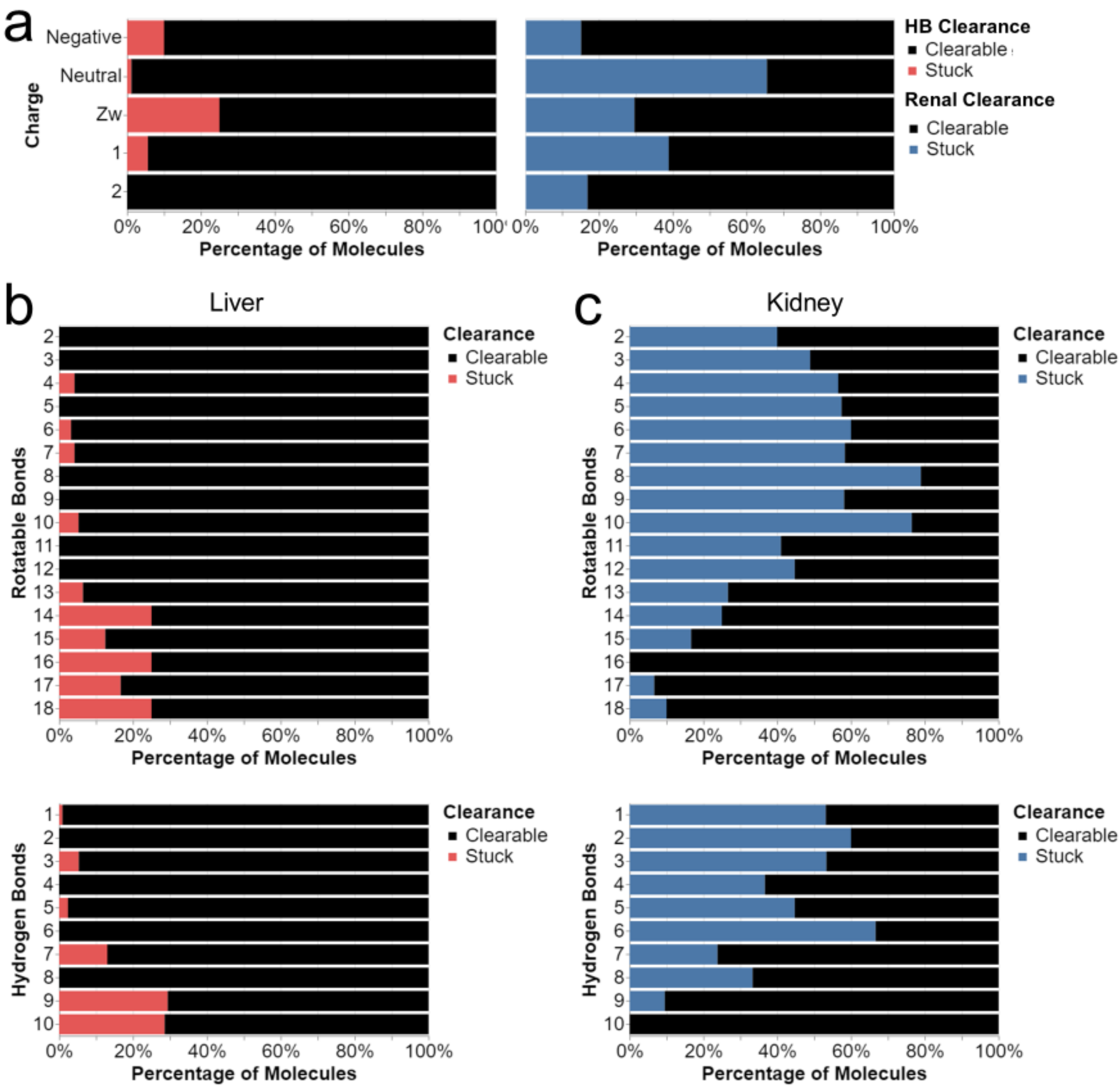

Figure S6. Effect of Polar Descriptors in Clearance. Using the clearance information from the database, the percentage of pentamethines and heptamethines that get stuck in the liver (a) and the kidney (b) for each category of net charge is shown. One positive charge is omitted for Zwitterionic molecules due to the indole nitrogen, negatively charged molecules are placed in "Negative," and those with positive net charges are categorized in their own respective charges. Rotatable bonds and hydrogen bonds (sum of hydrogen bond donors and acceptors) are presented up to a point due to the small number of molecules in the higher categories for the liver (b) and the kidney (c).

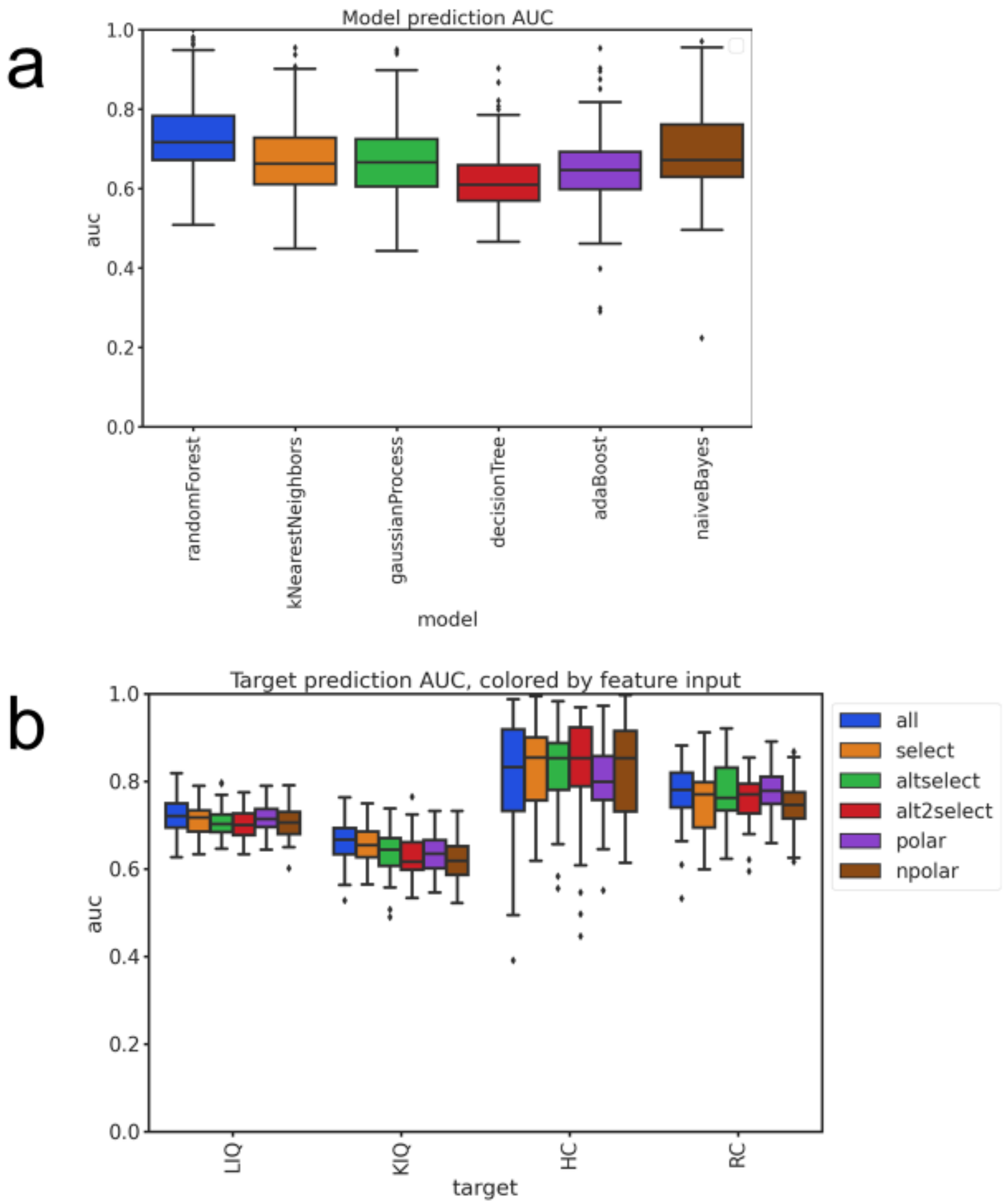

Figure S7. Machine Learning Results. (a) The box plot of AUC ROC scores of different machine learning algorithms, iterated $n = 3$ times over each target, feature, and structure inclusion. Overall, each algorithm showed an acceptable interquartile range of the AUC ROC score within 0.6 to 0.8. [9] (b) The box plot of AUC ROC scores of the baseline Random Forest algorithm, iterated $n = 15$ times over each feature and structure inclusion. Among the different targets, the hepatobiliary and renal clearance showed better results than the uptake quantile prediction, possibly due to binary classification resulting in a larger overall dataset than multiclass classification.

References

[1] C. R. Harris, K. J. Millman, S. J. van der Walt, R. Gommers, P. Virtanen, D. Cournapeau, E. Wieser, J. Taylor, S. Berg, N. J. Smith, R. Kern, M. Picus, S. Hoyer, M. H. van Kerkwijk, M. Brett, A. Haldane, J. F. del Río, M. Wiebe, P. Peterson, P. Gérard-Marchant, K. Sheppard, T. Reddy, W. Weckesser, H. Abbasi, C. Gohlke, T. E. Oliphant, *Nature* **2020**, *585*, 357-362.

[2] W. McKinney, in *Proceedings of the 9th Python in Science Conference, Vol. 445*, Austin, TX, **2010**, pp. 51-56.

[3] J. VanderPlas, B. Granger, J. Heer, D. Moritz, K. Wongsuphasawat, A. Satyanarayan, E. Lees, I. Timofeev, B. Welsh, S. Sievert, *J Open Source Softw* **2018**, *3*, 1057.

[4] F. Pedregosa, G. Varoquaux, A. Gramfort, V. Michel, B. Thirion, O. Grisel, M. Blondel, P. Prettenhofer, R. Weiss, V. Dubourg, *J Mach Learn Res* **2011**, *12*, 2825-2830.

[5] M. L. Waskom, *J Open Source Softw* **2021**, *6*, 3021.

[6] R. E. Schapire, *Empirical Inference*, Springer, **2013**, 37-52.

[7] G. T. Fechner, *Elemente der psychophysik, Vol. 2*, Breitkopf u. Härtel, **1860**.

[8] A. Matsui, E. Tanaka, H. S. Choi, V. Kianzad, S. Gioux, S. J. Lomnes, J. V. Frangioni, *Surgery* **2010**, *148*, 78-86.
